# 

**Published:** 1861-01

**Authors:** 


### MEDICAL CRITIC

AND

### PSYCHOLOGICAL

### JOURNAL.

EDITED BY

FORBES WINSLOW, M.D., D.C.L. OXON.

VOL. I.

S.G.O. LIBRARY
60771

LONDON:
JOHN W. DAVIES, 54, PRINCES STREET,
LEICESTER SQUARE.
EDINBURGH: MACLACHLAN AND CO. DUBLIN: FANNIN AND CO.

MDCCCLXI.

WI
ME286

LONDON:
SAVILL AND EDWARDS, PRINTERS, CHANDOS STREET,
COVENT GARDEN.

### NOTICE.

*There will be incorporated with the future numbers of the* "PSYCHOLOGICAL JOURNAL," *a series of Essays similar in literary character to the leading articles of* "THE TIMES" *and papers in the* "SATURDAY REVIEW," *illustrative of the* PRESENT *and* PROSPECTIVE *condition of the* MEDICAL PROFESSION *in its*

MORAL, POLITICAL,

SOCIAL, LITERARY,

AND

SCIENTIFIC

*relations.*

*The Journal is to be enlarged to two hundred pages, without increase of price. One hundred pages will be devoted to* PSYCHOLOGICAL *Subjects, and the remaining portion to Articles bearing upon general* MEDICAL POLITICS, LITERATURE, *and* SCIENCE.

*It will be the object of the Editor and his Literary Staff to discuss the important Questions of the day and Books that come under review in a fair, impartial, and liberal spirit, apart from feelings of a personal, private, or party character.*

